# Positive Interactions between Desert Granivores: Localized Facilitation of Harvester Ants by Kangaroo Rats

**DOI:** 10.1371/journal.pone.0030914

**Published:** 2012-02-14

**Authors:** Andrew J. Edelman

**Affiliations:** Department of Biology, University of New Mexico, Albuquerque, New Mexico, United States of America; University of Western Ontario, Canada

## Abstract

Facilitation, when one species enhances the environment or performance of another species, can be highly localized in space. While facilitation in plant communities has been intensely studied, the role of facilitation in shaping animal communities is less well understood. In the Chihuahuan Desert, both kangaroo rats and harvester ants depend on the abundant seeds of annual plants. Kangaroo rats, however, are hypothesized to facilitate harvester ants through soil disturbance and selective seed predation rather than competing with them. I used a spatially explicit approach to examine whether a positive or negative interaction exists between banner-tailed kangaroo rat (*Dipodomys spectabilis*) mounds and rough harvester ant (*Pogonomyrmex rugosus*) colonies. The presence of a scale-dependent interaction between mounds and colonies was tested by comparing fitted spatial point process models with and without interspecific effects. Also, the effect of proximity to a mound on colony mortality and spatial patterns of surviving colonies was examined. The spatial pattern of kangaroo rat mounds and harvester ant colonies was consistent with a positive interspecific interaction at small scales (<10 m). Mortality risk of vulnerable, recently founded harvester ant colonies was lower when located close to a kangaroo rat mound and proximity to a mound partly predicted the spatial pattern of surviving colonies. My findings support localized facilitation of harvester ants by kangaroo rats, likely mediated through ecosystem engineering and foraging effects on plant cover and composition. The scale-dependent effect of kangaroo rats on abiotic and biotic factors appears to result in greater founding and survivorship of young colonies near mounds. These results suggest that soil disturbance and foraging by rodents can have subtle impacts on the distribution and demography of other species.

## Introduction

Facilitation occurs when one species enhances the environment or performance of another species [1]. This positive interaction can arise directly through ecosystem engineering of the abiotic environment (e.g. solar radiation, water, or soil nutrients) or indirectly through effects on secondary species (e.g. suppressing a competitor or increasing abundance of prey) [2]. Facilitation in plant communities has been the focus of a considerable amount of ecological research [3], [4]. However, the role of facilitative interactions in shaping animal communities is less well understood [1]. Species interactions, such as facilitation, are usually scale dependent reflecting the spatial heterogeneity of abiotic and biotic factors [5], [6]. For example, the facilitative effects of nurse plants on seedling development are highly localized and diminish with distance [4]. Species interactions, along with other scale-dependent processes, affect population dynamics, which in turn influence the distribution of individuals across the landscape.

**Figure 1:**
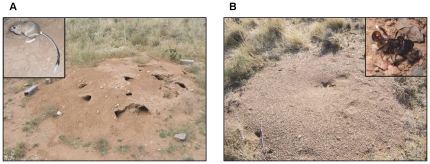
Representative structures built by banner-tailed kangaroo rats and rough harvester ants. Banner-tailed kangaroo rats build raised burrow systems called mounds (A) and rough harvester ant colonies build a cleared surface disc (B) over their underground nests. For scale, the live traps next to the mound are 30.5 cm long, whereas the colony disc is approximately 1 m in diameter.

In the Chihuahuan Desert, banner-tailed kangaroo rats (*Dipodomys spectabilis*) have keystone effects on community structure of plants, mammals, arthropods, and reptiles through their ecosystem engineering and selective seed predation [7], [8], [9], [10]. Banner-tailed kangaroo rats excavate large, semi-permanent mounds (Fig. 1A) that typically are 4 m in diameter and 30 cm in height and contain a labyrinth of tunnels and chambers extending up to 4 levels and >90 cm in depth [11], [12], [13]. This solitary species is highly territorial and vigorously defends its mound and the large caches of collected seeds contained within [13], [14]. New mounds are rarely built in established populations, rather mounds are occupied by subsequent generations of kangaroo rats [11], [15]. The soil disturbance and foraging activities of kangaroo rats are highly localized around mounds with the majority of their time spent within a 10-m radius of the mound [14].

Banner-tailed kangaroo rats are expected to strongly interact through exploitative resource competition with another granivore, the rough harvester ant (*Pogonomyrmex rugosus*), a dominant species of the Chihuahuan Desert ant community [16]. Similar to banner-tailed kangaroo rats, the activities of this colonial ant are localized around a central structure, an underground nest with a surface disc (Fig. 1B) cleared of vegetation and covered with small pebbles [17], [18]. Rough harvester ants forage for seeds both individually and in coordinated groups and store collected food in granaries within colonies [19], [20]. Differences in seed-size preferences and seasonal activity patterns likely reduce resource competition between banner-tailed kangaroo rats and rough harvester ants in the Chihuahuan Desert [21]. Banner-tailed kangaroo rats preferentially harvest seeds of large-seeded annuals, whereas rough harvester ants specialize on seeds of small-seeded annuals [22].

Instead of competing with rough harvester ants, banner-tailed kangaroo rats may facilitate ants through their effects on plant cover and seed resources. Soil moisture is an important factor in establishment and early survival of *P. rugosus* colonies [23]. Rough harvester ant foundresses (i.e. dispersing mated queens) almost exclusively initiate nests in open microhabitats rather than under vegetation, possibly because of reduced competition for soil moisture with perennial plants [24]. In addition, *P. rugosus* may conserve soil moisture by actively clearing vegetation and defoliating shrubs around the nest site [25]. Overall plant cover and perennial biomass are lower adjacent to banner-tailed kangaroo rat mounds [26], [27] possibly providing a greater density of suitable nest sites for *P. rugosus*. Seed resources are also likely more abundant near mounds which could enhance rough harvester ant populations. Biomass of small-seeded annuals, upon which rough harvester ants feed is greater within 4 m of mounds [26] and the soil surface at mounds has almost 7 times the seed density of off mound areas [28]. Despite these potential mechanisms of facilitation, removal experiments examining interactions between several species of kangaroo rats and harvester ants, have yielded only equivocal results, with no consistent effects on harvester ant populations when kangaroo rats are present [29].

The hypothesized relationship between banner-tailed kangaroo rats and rough harvester ants can be viewed as a scale-dependent interaction that decreases in strength with distance from centralized structures (i.e. mounds and colonies). Studying the spatial relationship between these species allows inference of whether and at what scale a positive or negative interaction occurs [6]. However, previous studies have not taken this type of spatially explicit approach, perhaps accounting for their difficulties in detecting an interaction [16], [21]. In this study, the potential interaction between kangaroo rats and harvester ants was examined as a point process, where stochastic intraspecific and interspecific spatial mechanisms (e.g. facilitation or competition) determined the point pattern (i.e. locations) of mounds and colonies across the landscape [6], [30]. I mapped the locations of mounds and colonies built by banner-tailed kangaroo rats and rough harvester ants, respectively, and monitored mortality and founding of ant colonies over two years. I used two spatially explicit techniques to explore the scale-dependent effects of kangaroo rats on ants. First, I compared the fit of statistical point process models to determine whether an interspecific interaction (positive or negative) characterized the spatial patterns of kangaroo rat mounds and established and recently founded colonies. Secondly, I used logistic regression models to examine whether colony mortality and the resulting spatial pattern of surviving colonies was spatially dependent on mounds. I predicted that mounds and colonies would exhibit a positive spatial interaction at small scales (<10 m) and colonies would have lower mortality risk near mounds due to the facilitative effects of soil disturbance and foraging by kangaroo rats. Analysis of spatial point patterns are typically limited to comparing observed spatial patterns to a null model based on complete spatial randomness to determine whether spatial aggregation or segregation occurs [6]. Statistical modeling of point patterns as done in this study, however, allows for more sophisticated hypothesis testing through fitting of observed spatial patterns to different proposed spatial interaction models and comparing the goodness-of-fit of observed data to simulated data from the best-fitting models [31]. These types of analyses have been used for examining interactions in plant communities [32], [33], but this study represents a novel application of them to animal communities.

## Methods

### Study area

The study area was located at the Sevilleta National Wildlife Refuge, near Socorro, New Mexico, USA (34°24′24.8′N, 106°36′20.5′W, 1600 m elevation). The site encompassed an 8.7-ha rectangular plot (397×220 m) of Chihuahuan Desert and short grass steppe vegetation dominated by grama grass (*Bouteloua eriopoda* and *B. gracilis*), burrograss (*Scleropogon brevifolius*), and sand dropseed grass (*Sporobolus cryptandrus*). This study was conducted under a research permit from the Sevilleta National Wildlife Refuge (Permit No. 07-020 and 08-032).

### Mapping and demography

A global positioning system (GeoXT, Trimble Navigation Ltd., Sunnyvale, California, USA) was used to map all banner-tailed kangaroo rat mounds and rough harvester ant colonies on the study site. Coordinates of structures were real-time differentially corrected using a local base station allowing for sub-meter accuracy of mapped locations.

The entire site was surveyed for *P. rugosus* colonies in June–September 2007 and again in August–September 2008. Colonies were located by walking 3-m transects across the study area and visually searching for signs of colonies. New colonies found in 2008 (classified as recently founded colonies) were assumed to have first been established by dispersing queens in summer 2007 and were not large enough to be detected until the following year [34]. Surveys were conducted during conditions of high ant activity (e.g. sunny weather and warm surface temperatures). Colonies were identified by the presence of a pebbled disc, nest entrance, and/or workers. All colonies were marked and the diameter of each colony disc was measured. In August 2008 and September–October 2009, all marked colonies were again checked for activity and the disc diameter was measured. Colonies were classified as active or inactive based on the presence of *P. rugosus*. In 2007 and 2008, inactive colonies were verified by disturbing the nest entrance to elicit an alarm response from workers [18]. In 2009, inactive colonies were also verified by digging into the disc and looking for workers. In addition, inactive colonies were revisited 1–2 weeks later and the verification process repeated. Inactive colonies were considered to have died between yearly surveys.

Mounds were located by walking adjacent 5-m transects throughout the study area during March 2005. From March 2005–February 2009 (excluding January 2007), a monthly mark-recapture census of the banner-tailed kangaroo rat population was performed on the study area to determine whether a mound was occupied. For a complete description of census methods and monthly occupancy criteria see [35]. All kangaroo rats were handled under methods approved by the University of New Mexico Institutional Animal Care and Use Committee (Protocol No. 04MCC00507 and UNM048-TR-100261). For comparison with ant colonies, a mound was considered occupied during a year if a resident kangaroo rat was present ≥1 month during a 12-month occupancy period. This criterion distinguished between mounds that were rarely occupied from those that were more frequently occupied, while accounting for any residual impacts of a kangaroo rat's soil disturbance and foraging activities. For 2007 and 2008, the occupancy period was July 2006–June 2007 and July 2007–June 2008, respectively. For 2009, the occupancy period was shorter, July 2008–February 2009, because monthly monitoring ended in February 2009. To compensate for the shorter occupancy period, all mounds were visually surveyed for signs of kangaroo rat activity in September 2009 and additional occupied mounds (*n* = 2) were included in the 2009 occupied group. Only occupied mounds were used in analyses because cessation of kangaroo rat activity leads to changes in plant communities around unoccupied mounds [26], [36].

### Spatial point process models

I used fitted spatial point process models to conduct formal hypothesis tests for the existence of an interspecific interaction between mounds and colonies [37]. The fits of two nested models were compared: (1) a reduced model that included intraspecific spatial interactions only, and (2) a full model that included both intraspecific and interspecific spatial interactions. Fitted full and reduced models were compared for two datasets: (1) all colonies and occupied mounds during 2007, and (2) occupied mounds and recently founded colonies during 2008. Recently founded colonies were defined as those discovered on the study area during 2008, whereas established colonies were first observed during 2007. Depending on the observed spatial patterns, the spatial interactions fitted in these models could be either symmetrical negative or positive in direction and vary in strength and scale.

All statistical models used were based on the Gibbs point process model, a flexible class of parametric statistical models that can include spatial interactions, spatial trends, and dependence on covariates [38]. These models can test for the existence of both negative and positive spatial interactions between locations. Gibbs models are specified in terms of conditional intensity, *λ*, which determines the conditional probability of finding a point at a given location based on information provided about the rest of the point process (e.g. interactions, covariates, and attributes) [37]. Specifically, the multi-type Strauss hard-core (MSHC) model with spatial covariates was chosen as the candidate model. This model allows for multiple discrete types of sites (e.g. mounds and colonies), hard-core properties (>1 structure cannot physically exist at the same location), covariates (e.g. distance to nearest established colony), and symmetrical positive and negative spatial interactions within and between species. See Information S1 for a detailed explanation of the MSHC model and model-fitting methods.

Intraspecific spatial interactions were included in the full and reduced MSHC models to control for the strong within-species competition of both species which results in uniform distributions of mounds and colonies through repulsion of conspecifics at short distances (≤30 m for kangaroo rats and ≤20 m for harvester ants) [18], [39]. A covariate based on the distance to nearest unoccupied mound was also included in all models because previous research indicated a negative interaction with occupied mounds [39], [40]. Because founding of harvester ant colonies is strongly affected by the presence of existing colonies [18], [34], a covariate based on distance to nearest established colony was added to models including recently founded colonies. A hard-core property for all interactions was also included in MSHC models, meaning that >1 mound or colony could not physically exist within an estimated distance of each other [18], [39].

All locations were marked as either kangaroo rat mounds or harvester ant colonies, denoted as *K* and *A* respectively. All MSHC models included parameters for intraspecific spatial interactions and spatial covariates (see Information S1 for detailed explanation of intraspecific parameters and covariates), but the parameters for the interspecific spatial interaction (*h_KA_*, *r_KA_*, and *γ_KA_*) were only included in the full models. The hard-core distance, *h_KA_*, specifies the radius around a location in which structures of different species cannot occur. The interaction distance, *r_KA_*, determines the radius around structures in which an interspecific interaction occurs and must be *>h_KA_*. The interaction parameter, *γ_KA_*, specifies the strength and direction of the interspecific interaction. For distances between *h_KA_* and *r_KA_* the interaction parameter is biologically interpreted as a positive interaction when *γ_KA_*>1 (i.e. attraction), no interaction if *γ_KA_* = 1, and a negative interaction if 0≤*γ_KA_<*1 (i.e. repulsion). The hard-core distances, interaction distances, and interaction parameters are all symmetric (e.g., *γ_KA = _γ_AK_*) [37]. I used the R package *spatstat* version 1.22-0 to fit MSHC models with conditional intensity estimated as a log-linear function. The model-fitting algorithm used a maximum pseudolikelihood method with a translation edge correction [41].

I examined the models' Akaike information criterion with second-order correction for small sample sizes (AIC_c_) and performed a Monte Carlo test with the log-pseudolikelihood ratio, *Δ*, as the test statistic to determine whether to reject the reduced model in favor of the full model (See Information S1 for calculation of *Δ*) [37]. If the reduced model was rejected (higher AIC_c_ and *P*<0.05 in Monte Carlo test), I then examined the interspecific parameters of the full model to interpret the biological significance of the spatial interaction between mounds and colonies (i.e. strength, direction, and scale of interaction). The goodness-of-fit of all models for each dataset was examined by comparing two complementary point pattern statistics, the bivariate modified K function (*L_KA_(r)*) and the refined nearest neighbor distances (*G_KA_(r)* and *G_AK_(r)*) of the observed spatial pattern to 95% critical envelopes based on 999 Monte Carlo simulations of the selected model. Translation and Kaplan-Meier edge corrections were used in calculation of the K function and refined nearest neighbor distances, respectively, at intervals of 0.1 m up to 20 m. Strong goodness-of-fit was characterized as a close match between spatial statistics of the observed spatial patterns and the mean of simulated patterns [37]. The K function totals the number of sites of the opposing species within a certain radius of a focal site and was used as an indicator of overall goodness-of-fit (see Information S1 for calculation of *L_KA_(r)*). The refined nearest neighbor distance is the cumulative frequency distribution of nearest neighbor distances and was calculated between sites of one species to the nearest neighboring opposing species sites [6]. The cumulative frequency distribution of nearest neighboring colony distances to mounds was expressed as *G_KA_(r)*, whereas nearest neighboring mound distances to colonies was expressed as *G_AK_(r)*. The refined nearest neighbor distances allowed detection of asymmetrical differences in goodness-of-fit between species. In a biological sense, *L_KA_(r)*, *G_KA_(r)*, and *G_AK_(r)* values above or below the 95% critical envelopes indicate that at those distances the observed locations were more aggregated or segregated, respectively, then predicted by the fitted model. I also used a modified version of the curvewise Cramer von Mises (CvM) statistic, the sum of the squared deviation of the observed spatial statistic from the expected value across all distances, to compare the goodness-of-fit between the full and reduced models. Because the expected value is unknown for MSHC models, I used the mean of the Monte Carlo simulations [33]. The model with the lowest CvM statistic was considered the best-fitting model.

### Mortality risk models

Mortality of harvester ant colonies is dependent on colony age, size, and neighborhood characteristics. Older colonies and larger colonies are less likely to die compared to recently founded and smaller colonies. Neighborhood characteristics such as high colony density can also increase mortality risk of colonies [18], [34], [42]. I used logistic regression to select variables that predicted mortality of recently founded and established colonies between 2008 and 2009 (period of highest mortality). Possible predictor variables that were examined for inclusion in mortality models were disc diameter and influence index. Influence index was a measure of competitive influence calculated as
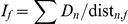
where *D_n_* is disc diameter of a neighboring colony, and dist*_n,f_* is the distance between a neighboring colony and focal colony [43]. Disc diameter was used as an index of colony size because it is positively correlated with number of workers in a colony [18], [44]. Due to age differences in foraging ranges [45], I calculated influence index for recently founded colonies at distances <10 m and for established colonies at distances <20 m. To determine the effect of kangaroo rat neighborhood characteristics on mortality risk, distance to nearest neighboring mound was included as a possible predictor variable. I examined the Akaike information criterion (AIC) of all possible model configurations to determine the best-fitting logistic regression model. Additionally, Wald tests were used to assess the significance of predictors in the best-fitting model. The model configuration with the lowest AIC was used in all further spatial analyses.

I tested whether the logistic mortality model or a random mortality model (i.e. null model) was a better predictor of the spatial patterns of surviving colonies in 2009. Each model was tested by comparing the univariate version of the homogeneous modified *K* function (*L(r)*) between observed and 95% critical envelopes generated by 999 Monte Carlo simulations of the logistic and random models. The univariate modified *K* function is calculated and interpreted similarly to the bivariate version except it only characterized the spatial relationship between colonies [6]. The modified *K* function was calculated at intervals of 0.1 m up to 20 m and a translation edge correction was used. Each simulation randomly thinned the 2008 point pattern based on an assigned probability of deletion. The resulting number of surviving colonies was the same as the number observed in 2009. Probability of deletion for the random mortality model was equal to the proportion of dead colonies in 2009 and the same for all colonies, whereas in the logistic mortality model probability of deletion for each colony was the fitted value of the logistic regression model [32]. Whichever model's simulated spatial pattern more closely matched the *L(r)* of the observed data was considered the better predictor of mortality risk. I also used the curvewise CvM statistic based on the mean of the Monte Carlo simulations to assess goodness-of-fit between mortality models. I used *spatstat* to perform spatial analyses and the R package *ecespa* version 1.1-04 to compute the critical envelopes of *L(r)* from mortality models [46].

## Results

### Demography

Colony size and mortality rate varied between years and colony type (Table 1). The number of colonies detected on the site increased by 77% from 2007–2008. Overall colony mortality was very low during 2007–2008 (4%), but increased substantially during 2008–2009 (19%). The mortality rate for recently founded colonies was almost 3 times higher than for established colonies during 2008–2009 (Pearson's chi-square test, *χ_2_* = 24.7, *P*<0.0001). Established colonies had a significantly larger disc diameter than recently founded colonies during both 2008 and 2009 (two-tailed *t*-test, all *P*<0.0001). Disc diameter increased with age (paired two-tailed *t*-tests, all *P*<0.001) for both established (2008: mean ± SE = 18.6±1.7 cm; 2009: mean ± SE = 11.9±1.5 cm) and recently founded colonies (mean ± SE = 13.1±2.0 cm). The number of occupied kangaroo rat mounds varied slightly between years: 48 in 2007, 44 in 2008, and 42 in 2009. In total, 56 different mounds were occupied from 2007–2009. The majority of mounds were occupied all three years (65%) and at least two out of three years (84%).

**Table 1:** Demography of rough harvester ant colonies from 2007–2009.

Year	Colony type	*n*	Disc diameter (cm)^a^	% Mortality/year (*n*)
2007	All colonies	212	89.3±2.8	
2008	Established colonies^b^	204	109.4±2.9	4% (8)
	Recently founded colonies^c^	162	33.3±2.7	
2009	Established colonies^b^	184	126.6±2.8	11% (20)
	Recently founded colonies^c^	113	54.0±3.9	43% (49)

a Mean ± SE.

b Surviving colonies marked in 2007.

c Colonies first detected in 2008.

### Interspecific spatial patterns

**Figure 2:**
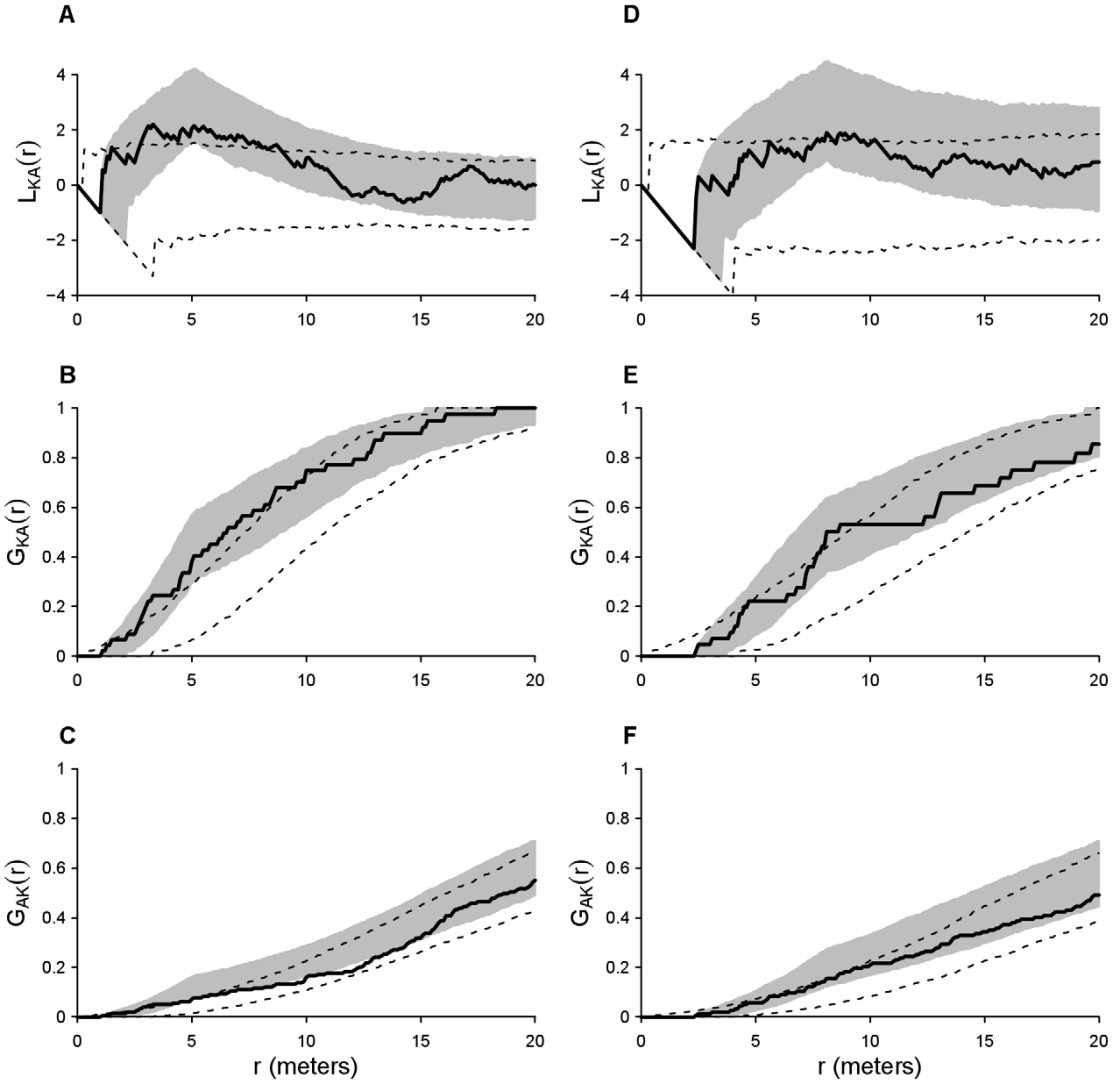
Goodness-of-fit for spatial point process models of mound and colony distributions. Comparison of the spatial statistics *L_KA_(r)*, *G_KA_(r)*, and *G_AK_(r)* for the observed spatial patterns and spatial patterns simulated from fitted full models containing an interspecific interaction and reduced models without an interspecific interaction. Goodness-of-fit for all occupied kangaroo rat mounds (*n* = 48) and harvester ant colonies (*n* = 212) during 2007 are shown in panels A, B, and C and for all occupied kangaroo rat mounds (*n* = 44) and recently founded harvester ant colonies (*n* = 162) during 2008 are shown in panels D, E, and F. Solid black lines represent the observed spatial statistics (*L_KA_(r)*, *G_KA_(r)*, and *G_AK_(r))*. Dashed lines and shaded areas denote the 95% critical envelopes of spatial statistics generated from 999 Monte Carlo simulations of the reduced and full models, respectively.

Best-fitting spatial models supported the presence of a small-scale positive interaction between occupied mounds and both existing and recently founded colonies. In 2007, the full model with a positive interspecific interaction included (log-pseudolikelihood = −1755.5, AIC_c_ = 3469.0, w_i_ = 1) was a significantly better fit than the reduced model (log-pseudolikelihood = −1780.7, AIC_c_ = 3489.4, w_i_ = 0) for the spatial pattern of mounds and established colonies even after controlling for intraspecific interactions (*Δ* = 50.4, *P* = 0.001). Based on the full model's interspecific parameters, mounds and established colonies were 3.7 times more likely to occur at scales of 1–5.1 m around opposing species sites than at larger scales (*h_KA_* = 1 m, *r_KA_* = 5.1 m, *γ_KA_* = 3.7; see Table S1 for all model parameters). In 2008, the full model with a positive interspecific interaction (log-pseudolikelihood = −1477.3, AIC_c_ = 2927.6, w_i_ = 1) was also a better fit than the reduced model (log-pseudolikelihood = −1498.1, AIC_c_ = 2964.2, w_i_ = 0) for the spatial pattern of mounds and recently founded colonies even after controlling for effects of intraspecific interactions (*Δ* = 41.6, *P* = 0.003). Mounds and recently founded colonies were 2.3 times more likely to occur at scales of 2.3–8.1 m around opposing species sites (*h_KA_* = 2.3 m, *r_KA_* = 8.1 m, *γ_KA_* = 2.3; see Table S1 for all model parameters). Comparison of observed values of spatial statistics (*L_KA_(r)*, *G_KA_(r)*, and *G_AK_(r)*) to 95% critical envelopes for both 2007 (Fig. 2A–C) and 2008 point patterns (Fig. 2D–F) confirmed that the simulated spatial patterns from the full models closely matched the observed spatial patterns. The observed *L_KA_(r)* and *G_KA_(r)* values remained well within the 95% critical envelopes and exhibited similar interspecific aggregation (*L_KA_(r)*>0) at small scales as the mean full model simulations values. Only observed *G_AK_(r)* values in 2007 showed any significant deviation from the full model simulations with lower values than the 95% critical envelopes between 5–14 m. The curvewise CvM statistics also supported that the full model was generally a better fit to the observed spatial patterns than the reduced model. In both years, the CvM statistics of the full model were lower than the reduced model for both *L_KA_(r)* (2007: full model = 56.9, reduced model = 289.2; 2008: full model = 81.1, reduced model = 290.8) and *G_KA_(r)* (2007: full model = 0.1, reduced model = 2.7; 2008: full model = 0.9, reduced model = 1.1). The CvM statistics for *G_AK_(r)* in 2007 (full model = 0.7, reduced model = 0.1) and 2008 (full model = 0.6, reduced model = 0.2) indicated that the reduced model was a better fit.

### Colony mortality risk

**Figure 3:**
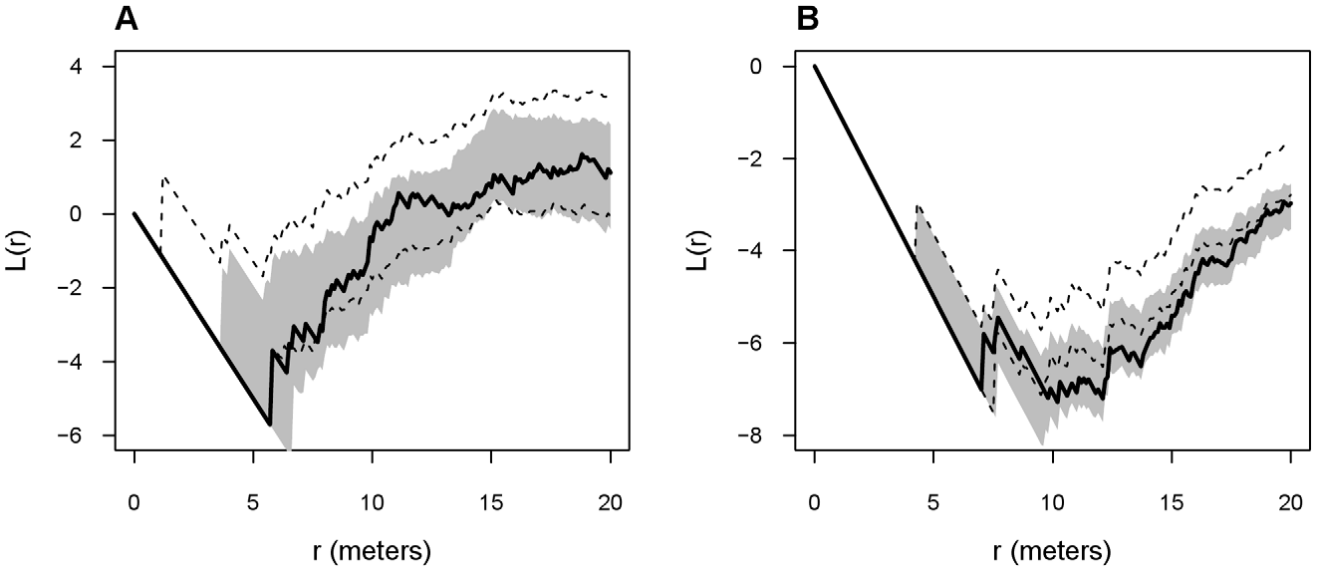
Goodness-of-fit for colony mortality risk spatial models. Comparison of the spatial statistic *L(r)* from observed spatial patterns and spatial patterns simulated from mortality risk models for surviving A) recently founded ant colonies and B) established ant colonies during 2009. Solid black lines represent the observed *L(r)*. Dashed lines and shaded areas are the 95% critical envelopes of *L(r)* generated from 999 Monte Carlo simulations of the random mortality model and logistic mortality model, respectively.

Recently founded colonies had lower mortality risk near kangaroo rat mounds. Three parameters, including one based on spatial relationship to neighboring mounds, were selected for inclusion in the final logistic regression model of recently founded colony mortality risk from 2008–2009 (Table 2; log-likelihood = −81.7, AIC = 171.4, w_i_ = 0.79). Mortality risk of recently founded colonies decreased as disc diameter increased and distance to nearest mound and influence index decreased. The observed locations of surviving recently founded colonies in 2009 were most consistent with the logistic mortality model (CvM statistic: logistic model = 113.1, random model = 323.7). The observed *L(r)* function was completely enclosed by the 95% critical envelopes generated by the logistic mortality model (Fig. 3A, shaded area). The simulated spatial patterns of the logistic mortality model exhibited similar levels of small-scale regularity between colonies (*L(r)*<0) as the observed spatial pattern. In addition, simulations of the logistic mortality model resulted in a small-scale hard-core property (i.e. minimum distance between colonies) between approximately 4–6 m, similar to the observed spatial pattern. The random mortality model was a poorer predictor of the observed spatial pattern of surviving recently founded colonies in 2009, particularly at small scales (Fig. 3A, dashed lines). The observed *L(r)* function exceeded the 95% critical envelopes generated by the random mortality model several times between 5–7 m and failed to develop a consistent hard-core property. The *L(r)* function of the random mortality model simulations also tended to produce weaker regularity between colonies than the observed spatial pattern.

**Table 2:** Best-fitting logistic regression models of 2008–2009 mortality risk for recently founded (*n* = 162) and established rough harvester ant colonies (*n* = 204).

Colony type	Model parameters	Coefficient	SE	*P*
Recently founded colonies	Intercept	1.275	0.451	0.0047
	Disc diameter	−0.029	0.008	0.0002
	10-m Influence index	0.073	0.027	0.0067
	Nearest mound distance	0.037	0.017	0.0271
Established colonies	Intercept	1.045	0.866	0.23
	Disc diameter	−0.025	0.006	<0.0001
	20-m Influence index	0.045	0.022	0.044

Mortality risk of established colonies was not affected by kangaroo rat mounds. Two parameters were selected as predictors of 2008–2009 mortality risk for established colonies, however, no variables related to neighboring mounds were selected (Table 2; log-likelihood = −49.8, AIC = 105.6, w_i_ = 0.48). Mortality risk of established colonies decreased as disc diameter increased and influence index decreased. The logistic mortality model was a better predictor of the spatial patterns of surviving established colonies in 2009 than the random mortality model (Fig. 3B; CvM statistic: logistic model = 21.9, random model = 184.5). The observed *L(r)* function was completely enclosed by the 95% critical envelopes of the logistic mortality model over all distances (Fig. 3B, shaded area), whereas it exceeded the envelopes of the random mortality model at distances >10 m (Fig. 3B, dashed lines). The simulations of the random mortality model produced weaker regularity between colonies, then the observed spatial pattern and the logistic mortality model.

## Discussion

My results provide evidence of a localized positive interaction such as facilitation between banner-tailed kangaroo rats and rough harvester ants in the Chihuahuan Desert. Best-fitting spatial models contained a positive interspecific interaction at small scales for both established and recently founded colonies (Fig. 2A–F). The best-fitting mortality risk model for recently founded colonies also accurately described spatial patterns of surviving colonies and indicated that survivorship increased with decreasing distance from a kangaroo rat mound (Fig. 3A). These spatial patterns and colony dynamics may be related to the benefits of being close to areas of high kangaroo rat activity.

**Figure 4:**
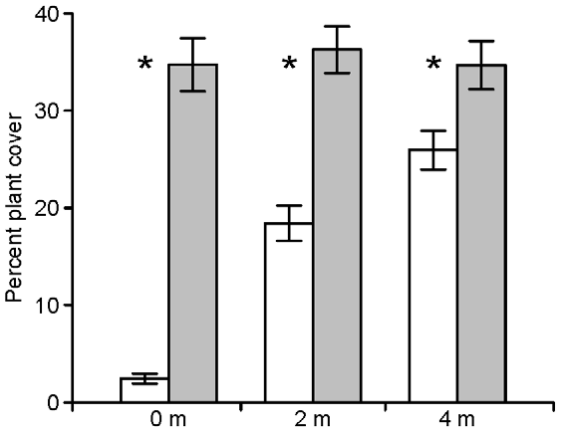
Patterns of vegetation cover at kangaroo rat mounds. Percent plant cover (mean ± SE) in 1-m quadrats at occupied banner-tailed kangaroo rat mounds (white bars, *n* = 30) and random sites >20 m from a mound (shaded bars, *n* = 30). Percent plant cover was measured at 0 m, 2 m, and 4 m from center of the mound or random site. Vegetation was surveyed twice during summer 2006 and averaged for each site. An asterisk denotes a statistically significant difference (*P<0*.*05* in two-tailed *t*-test on arcsine square root transformed data) between mounds and random sites at that distance.

The most parsimonious mechanism for the observed spatial patterns is the localized facilitation of rough harvester ants by banner-tailed kangaroo rats, perhaps mediated via effects on plant cover and composition. Kangaroo rats actively reduce total plant cover on and immediately adjacent to their mounds [26], [27], [28], which may attract ant foundresses and improve colony survivorship. I also observed a strong decrease in vegetative cover around mounds at my study area compared to random sites (Fig. 4; A.J. Edelman and S. Whiteman, unpublished data). Lower plant cover may enhance founding and early survival of ant colonies. Rough harvester ant foundresses select open microhabitats for nest sites, a characteristic that may be linked to higher soil moisture conditions [24], [25]. Soil moisture is an important factor in early colony survivorship and open microhabitats may reduce competition for this resource with perennial plants [23], [24]. Kangaroo rats also increase the abundance of harvester ants preferred food source, small-seeded annuals, by selectively foraging on competitively superior large-seeded annuals and creating disturbed habitats which favor small-seeded annuals [16], [21], [47], [48]. As a result, biomass and seeds of small-seeded annuals are more abundant on and near banner-tailed kangaroo rat mounds than inter-mound areas [26], [28], [49]. I also observed greater numbers of seeds in the soil surface near rough harvester colonies that were proximate to mounds, than those farther away. Seed abundance in soil surface samples from my study area (1×12.5×12.5-cm samples taken 5 m from colony in a random direction) was 3 times greater (two-tailed *t*-test on log-transformed data, *t_28_* = 2.7, *P* = 0.013) at colonies <10 m from occupied mounds (mean ± SE = 101.4±24.3 seeds, *n* = 15) than colonies located >20 m away (mean ± SE = 35.9±5.9 seeds, *n* = 15) (A. J. Edelman and E. Tuttle, unpublished data). Whether ant colonies near mounds actually benefit from access to greater seed resources is unclear and requires further study. Preliminary research suggests that *P. rugosus* workers are more common around mounds. Abundance of *P. rugosus* workers captured in pitfall traps on my study area (two traps per site opened 20 days during June–July 2007) was 4 times greater (two-tailed *t*-test on log-transformed data, *t_28_* = 2.7, *P* = 0.011) at occupied mounds (mean ± SE = 79.7±36.3 workers, *n* = 15) than at random sites >20 m from a mound (mean ± SE = 20.1±5.6 workers, *n* = 15) (A. J. Edelman and S. Johnson, unpublished data).

Any interspecific interaction is likely to be asymmetric, with kangaroo rats affecting the spatial pattern of harvester ant colonies, but not vice versa. In the Chihuahuan Desert, no dramatic changes in plant communities or rodent populations were detected when harvester ants were removed [16], [21]. While founding of new harvester ant colonies is common [18], new kangaroo rat mounds are rarely built in established populations. Instead, mounds are occupied by different individuals over many generations and can exist for >50 years [11], [15]. Total number and identity of occupied mounds varied little over the study period in comparison to colonies. The MSHC models could only include symmetric interactions, however, the goodness-of-fit of the observed data to the model simulations revealed possible interaction asymmetries between species (Fig. 2). While the cumulative frequency distribution of distances from mounds to nearest neighboring ant colonies (*G_KA_(r)*) closely matched the simulation mean (Fig. 2B and E), the cumulative frequency distribution of distances from colonies to nearest neighboring mounds (*G_AK_(r)*; Fig. 2C and F) tended to be lower than expected (i.e. mounds were less aggregated around colonies than predicted by the fitted models). This discrepancy between species indicates that colonies may have a weaker positive or nonexistent effect on mound spatial patterns.

The potential effect of kangaroo rats on harvester ants appears to be greatest when colonies are young and highly vulnerable to mortality. Young *Pogonomyrmex* colonies often have a mortality rate from colony establishment to 2 years of age near 99% [34], [50]. In my study, considerable mortality of recently founded colonies had likely already occurred by the time these colonies were first detected in 2008. These same colonies also experienced additional high mortality (>40%) over the following year. During this vulnerable period, close proximity to a kangaroo rat mound may have provided an advantage, contributing to the higher occurrence probability and lower mortality risk near mounds. Older colonies have likely gone through the same founding and mortality pressures as experienced by younger colonies because they had an even stronger probability of occurring near mounds than recently founded colonies. However, established harvester ant colonies had low mortality and proximity to a mound did not have an effect on short-term survivorship. Further research is necessary to determine if mounds may affect long-term survivorship or reproductive success of established colonies.

The potential interaction between kangaroo rats and harvester ants is highly localized on the landscape. Only an estimated 17% of the study area was affected by this interaction (based on 48 occupied mounds with a 10-m radius of influence). Intraspecific competition among colonies and mounds is undoubtedly a stronger interaction than facilitation and results in the uniform distribution of these structures across the landscape [18], [39]. However, intraspecific competition alone was not able to account for the spatial patterns of colonies and mounds. MSHC and logistic models containing both intraspecific and interspecific interactions were a better fit to the observed data than models with only intraspecific interactions. Some observed founding and mortality events may have actually been colonies that moved locations, perhaps to reduce competition with other colonies or move closer to a mound. In *P. barbatus*, no more than 10% of colonies relocate per year [51]. Even if a subset of colonies relocated, the underlying implications remain unchanged. Colonies may be more likely to occur near kangaroo rat mounds through dynamics of founding, mortality, and perhaps even relocation.

The complexity and strength of interactions between harvester ants and kangaroo rats vary between desert regions in the American Southwest [29]. In the Sonoran Desert, both exploitative competition and facilitation likely exist between kangaroo rats and harvester ants because experimental removal of kangaroo rats in the Sonoran Desert resulted in short-term increases in ant abundance, but long-term decreases [52], [53]. Competition may play a lesser role in the Chihuahuan desert, because of differences in seed preferences, activity patterns, and seasonality in resources between kangaroo rats and harvester ants [16], [21], [48], [54]. My results support that banner-tailed kangaroo rats and rough harvester ants do not strongly compete for resources in the Chihuahuan Desert. None of the best-fitting spatial models included a negative interspecific interaction parameter (e.g. *γ_KA_*<1) as expected if strong competition existed between species. Furthermore, the logistic mortality model indicated a positive rather than negative spatial influence of mounds on recently founded colony survival.

Spatial point pattern analysis has rarely been applied to the study of animal community structure and typically only inductive pattern analysis has been used. This study uses a model comparison approach to examine species relationships based on statistical point process modeling of interactions and mortality spatial patterns. As such, it demonstrates the usefulness of these techniques in teasing apart scale-dependent processes in animal communities. Previous experimental research failed to detect a positive interaction between kangaroo rats and harvester ants [16], [29] possibly because scale was not explicitly included in analyses.

Banner-tailed kangaroo rats have well documented impacts on community structure of plants and animals in the American Southwest [7], [8], [9], [10]. My study reveals the potential for a localized positive effect on the spatial pattern and dynamics of rough harvester ants. Facilitation between animals is likely common in communities, but has received little attention from ecologists compared to facilitation among plants [1], [3], [4]. Identifying the influence of these positive interactions provides a more comprehensive understanding of natural communities and aids in their conservation [2].

## Supporting Information

Information S1 **Spatial point process modeling methods.**
(DOC)

Table S1 Parameters of best-fitting multi-type Strauss hard-core models for spatial point patterns of banner-tailed kangaroo rat mounds and rough harvester ant colonies (See Information S1 for descriptions of model parameters).
(DOC)
