# Supplementary material for: Positive Interactions between Desert Granivores: Localized Facilitation of Harvester Ants by Kangaroo Rats
Source: PLoS One. 2012 Feb 14;7(2):e30914. doi: 10.1371/journal.pone.0030914 (PMC3279350; doi:10.1371/journal.pone.0030914)
Supplement: Information S1 — Spatial point process modeling methods. (DOC) [file pone.0030914.s001.doc]

Information S1. Spatial point process modeling methods

Multi-type Strauss hard-core model

The multi-type Strauss hard-core (MSHC) model allows for multiple discrete types of sites (e.g. mounds and colonies), hard-core properties (> 1 structure cannot physically exist at the same location), and both symmetrical positive and negative spatial dependence within and between species. Let y = {(*x_1_, m_1_*),…, (*x_n_, m_n_*)} denote a multi-type point pattern of a point process in a bounded region *W* R^2^ with a set of *M* possible species, where *x_i_ $\in$W* are the sites and *m_i_ $\in$M* the corresponding species labels. The MSHC model with spatial covariates where *u* $\in$ *W* and *k* $\in$ *M*, has a conditional intensity function of

$$\lambda\left(\left(u,k\right),y\right)=B_{k}\left(u\right)\prod_{i} g\left(k,m_{i},\left\|u-x_{i}\right\|\right)$$

where the interaction between a pair of sites *u* and *x* of species *k* and *m* is determined by the function

The four main parameters that define the MSHC model are *B_k_(u)*, *h_km_*, *r_km_,* and *γ_km_* (all must be > 0). Function *B_k_(u)* determines the intensity of the process for each species and includes spatial trends and dependence on covariates at point *u*. The hard-core distance, *h_km_*, specifies the radius around a site in which other structures cannot occur. The interaction distance, *r_km_*, determines the radius around structures in which a spatial interaction occurs and must be *> h_km_*. The interaction parameter, *γ_km_*, specifies the strength and direction of the interaction. For distances between *h_km_* and *r_km_* the interaction parameter is biologically interpreted as a positive interaction when *γ_km_* > 1 (i.e. attraction), no interaction if *γ_km_* = 1, and a negative interaction if 0 ≤ *γ_km_ <* 1 (i.e. repulsion). The hard-core distances, interaction distances, and interaction parameters are all symmetric (e.g. *γ_km =_ γ_mk_*) .

Model-fitting methods

All locations were marked as either kangaroo rat mounds or harvester ant colonies, denoted as *K* and *A* respectively. The full MSHC model had 11 total parameters; 5 regular parameters (*B_K_(u), B_A_(u), γ_KK_, γ_AA_,* and *γ_KA_*) and 6 irregular parameters (*h_KK_, h_AA_, h_KA_, r_KK_*, *r_AA_*, and *r_KA_*). Irregular parameters were estimated using the maximum-likelihood and maximum-pseudolikelihood methods outlined below. Regular parameters were estimated as part of the model-fitting algorithm. Depending on the dataset, functions *B_K_(u)* and *B_A_(u)* included 1–2 spatial covariates. As a result, these functions took the log-linear form

$$B_{k}\left(u\right)=\text{exp}\left(\beta_{0}+\beta_{1}S\left(u\right)+\beta_{2}Z\left(u\right)\right)$$

where the coefficient parameters unique to each mark are *β_0_*, *β_1_*, and *β_2_* and *S(u)* and *Z(u)* are the covariates at point *u*. A covariate based on the distance to nearest unoccupied mound, *S(u)*, was included in all models because previous research indicated a negative interaction with occupied mounds . Because founding of harvester ant colonies is strongly affected by the presence of existing colonies , I added a covariate based on distance to nearest established colony, *Z(u),* to models fitted to datasets including recently founded colonies. I created covariates by dividing the entire study area into 1 × 1-m pixels and assigning pixel values as the distance from each pixel to the nearest relevant site (e.g. unoccupied mound or established colony).

I used the maximum likelihood method to estimate hard-core distances, which corresponded to minimum interpoint distances (e.g. minimum observed distance between ant colonies). I used the profile pseudolikelihood method with a border edge correction to estimate interaction distances . This method found the interaction distance with the maximum pseudolikelihood between the hard-core distance and a set maximum distance in steps of 0.1 m. The upper distance limit in profile pseudolikelihood method was set as 30 m and 20 m for intraspecific interactions of kangaroo rats and harvester ants, respectively, and 20 m for interspecific interactions. These maximum distances were selected based on the hypothesized scale of interactions within and between species.

I performed a Monte Carlo test with the log-pseudolikelihood ratio, *Δ*, as the test statistic to determine whether to reject the reduced model . The Metropolis-Hastings algorithm with 100,000 iterations was implemented to generate 999 simulations of the reduced model. For each simulation, I fitted the full and reduced models and calculated *Δ* as twice the log pseudolikelihood ratio. Finally, the *P* value of the Monte Carlo test was determined by ranking the observed *Δ* in the set of simulation *Δ*’s.

Calculation of bivariate modified K function

The bivariate *K* function totals the number of sites of the opposing species within a radius *r* of a focal site (mound or colony):

$K_{12}\left(r\right)=A\sum_{\begin{matrix} i=1 \\ i\neq j \end{matrix}}^{n_{1}} \sum_{\begin{matrix} j=1 \\ j\neq i \end{matrix}}^{n_{2}} \frac{\left(w_{ij}\cdot d_{ij}\left(r\right)\right)}{n_{1}n_{2}}$

where $K_{12}\left(r\right)$ is the observed estimate *K* at distance *r*, *A* is the area of the study plot, *n_1_* and *n_2_* are the number of sites of each species, *w_ij_* is the edge-correction weighting, and *d_ij_(r)* is the distance between sites *i* and *j* and *d_ij_(r)* = 1 if *d_ij_ ≤ r* and *d_ij_(r) = 0 if d_ij_ > r* . Bivariate *K* was calculated for each species separately and combined into one estimate. For ease of interpretation and to stabilize the variance, I used a modified *K* function, $L_{12}\left(r\right)$:

$L_{12}\left(r\right)=\left(\mathop{K}\limits^{\wedge} _{12}\left(r\right)/\pi\right)^{1/2}-r$

Biologically, $L_{12}\left(r\right)$ for two point patterns can be interpreted as exhibiting, with respect to each other, complete spatial randomness when zero, aggregation when > 0, and segregation when < 0 . $L_{12}\left(r\right)$ was calculated for distances ≤ 20 m at 0.1-m intervals and a translation edge correction was implemented.

References

1. Baddeley A, Turner R (2006) Modelling spatial point patterns in R. In: Baddeley A, Gregori P, Mateu J, Stoica R, Stoyan D, editors. Case studies in spatial point pattern modelling. New York: Springer-Verlag. pp. 23–74.

2. Schroder GD, Geluso KN (1975) Spatial distribution of *Dipodomys spectabilis* mounds. Journal of Mammalogy 56: 363–368.

3. Schooley RL, Wiens JA (2001) Dispersion of kangaroo rat mounds at multiple scales in New Mexico, USA. Landscape Ecology 16: 267–277.

4. Gordon DM, Kulig AW (1996) Founding, foraging, and fighting: colony size and the spatial distribution of harvester ant nests. Ecology 77: 2393–2409.

5. Schooley RL, Wiens JA (2003) Spatial patterns, density dependence, and demography in the harvester ant, *Pogonomyrmex rugosus* in semi-arid grasslands. Journal of Arid Environments 53: 183–196.

6. Baddeley A, Turner R (2005) Spatstat: an R package for analyzing spatial point patterns. Journal of Statistical Software 12: 1–42.

7. Ripley BD (1977) Modelling spatial patterns. Journal of the Royal Statistical Society Series B (Statistical Methodology) 39: 172–212.

8. Fortin M-J, Dale MRT (2005) Spatial analysis: a guide for ecologists. Cambridge: Cambridge University Press. 365 p.
