# Supplementary material for: Positive Interactions between Desert Granivores: Localized Facilitation of Harvester Ants by Kangaroo Rats
Source: PLoS One. 2012 Feb 14;7(2):e30914. doi: 10.1371/journal.pone.0030914 (PMC3279350; doi:10.1371/journal.pone.0030914)
Supplement: Table S1 — Parameters of best-fitting multi-type Strauss hard-core models for spatial point patterns of banner-tailed kangaroo rat mounds and rough harvester ant colonies (See Information S1 for descriptions of model parameters). (DOC) [file pone.0030914.s002.doc]

**Table S1.** Parameters of best-fitting multi-type Strauss hard-core models for spatial point patterns of banner-tailed kangaroo rat mounds and rough harvester ant colonies (See Information S1 for descriptions of model parameters).

| Model parameters | Mounds | Colonies | Interspecific interaction |
| --- | --- | --- | --- |
| *2007: all colonies and occupied mounds* |  |  |  |
| *n* | 48 | 212 |  |
| *B_k_(u)=* exp(*β_0_ + β_1_S(u))* (points/m^2^)^a^ | 0.0010 | 0.0055 |  |
| *β_0_* | -7.91 | -5.0 |  |
| *β_1_* | 0.03 | -0.007 |  |
| *h* (m) | 14.9 | 1.7 | 1.0 |
| *r* (m) | 21.2 | 12.1 | 5.1 |
| *γ* | 0.25 | 0.26 | 3.65 |
|  |  |  |  |
| *2008: recently founded colonies and occupied mounds* | |  |  |
| *n* | 44 | 162 |  |
| *B_k_(u)=* exp(*β_0_ + β_1_S(u)* + *β_2_Z(u))* (points/m^2^)^a^ | 0.00085 | 0.0023 |  |
| *β_0_* | -7.87 | -8.08 |  |
| *β_1_* | -0.07 | 0.14 |  |
| *β_2_* | 0.04 | 0.01 |  |
| *h* (m) | 10.6 | 1.1 | 2.3 |
| *r* (m) | 19.7 | 9.5 | 8.1 |
| *γ* | 0.2 | 0.8 | 2.3 |

^a^Covariates based on distance from unoccupied mounds (*S(u)*) and established colonies (*Z(u)*) were set to mean species value for calculations of *B_k_(u)*.
